# Nucleotide exchange factor Rab3GEP requires DENN and non-DENN elements for activation and targeting of Rab27a

**DOI:** 10.1242/jcs.212035

**Published:** 2019-04-30

**Authors:** Paolo Sanzà, Richard D. Evans, Deborah A. Briggs, Marta Cantero, Lluis Montoliu, Shyamal Patel, Elena V. Sviderskaya, Aymelt Itzen, Ana C. Figueiredo, Miguel C. Seabra, Alistair N. Hume

**Affiliations:** 1 School of Life Sciences, University of Nottingham, Nottingham NG7 2UH, UK; 2 Centro Nacional de Biotecnologia (CNB-CSIC), Madrid 28049, Spain; 3 CIBERER-ISCIII, Madrid 28029, Spain; 4 Cell Biology and Genetics Research Centre, Molecular and Clinical Sciences Research Institute, St. George's, University of London, London SW17 0RE, UK; 5 Center for Integrated Protein Science Munich (CIPSM), Department of Chemistry, Technische Universität München, Garching 85748, Germany; 6 Instituto de Biologia Molecular e Celular, Universidade do Porto, Porto 4200-135, Portugal; 7 i3S-Instituto de Investigação e Inovação em Saúde, Universidade do Porto, Porto 4200-135, Portugal; 8 CEDOC Faculdade de Ciencias Medicas, Universidade Nova de Lisboa, Lisbon 1169-056, Portugal

**Keywords:** Organelle transport, Guanine nucleotide exchange factor, Rab27a, Rab3GEP, MADD, Melanocyte

## Abstract

Rab GTPases are compartment-specific molecular switches that regulate intracellular vesicular transport in eukaryotes. GDP/GTP exchange factors (GEFs) control Rab activation, and current models propose that localised and regulated GEF activity is important in targeting Rabs to specific membranes. Here, we investigated the mechanism of GEF function using the Rab27a GEF, Rab3GEP (also known as MADD), in melanocytes as a model. We show that Rab3GEP-deficient melanocytes (melan-R3G^KO^) manifest partial disruption of melanosome dispersion, a read-out of Rab27a activation and targeting. Using rescue of melanosome dispersion in melan-R3G^KO^ cells and effector pull-down approaches we show that the DENN domain of Rab3GEP (conserved among RabGEFs) is necessary, but insufficient, for its cellular function and GEF activity. Finally, using a mitochondrial re-targeting strategy, we show that Rab3GEP can target Rab27a to specific membranes in a GEF-dependent manner. We conclude that Rab3GEP facilitates the activation and targeting of Rab27a to specific membranes, but that it differs from other DENN-containing RabGEFs in requiring DENN and non-DENN elements for both of these activities and by lacking compartment-specific localisation.

## Abstract (precis)

**Summary:** Rab3GEP plays an important, but not exclusive, role in organelle targeting of Rab27a, and the Rab3GEP DENN domain alone is insufficient to activate and target Rab27a.

## INTRODUCTION

Rab proteins are a family (>60 in humans) of small GTPases that regulate vesicle trafficking in eukaryotic cells (Stenmark, 2009; Hutagalung and Novick, 2011). Compartment-specific localisation is vital for Rab function, but the mechanism(s) regulating this remain debatable (Barr, 2013; Pfeffer, 2017). Recent studies indicate that localised GDP/GTP exchange factors (GEFs) contribute to activation and targeting of Rabs (Allaire et al., 2010; Yoshimura et al., 2010; Ingmundson et al., 2007; Machner and Isberg, 2006; Murata et al., 2006; Tarafder et al., 2011; Zhang et al., 2006). Two studies have directly investigated this by showing that substrate Rabs follow artificially targeting GEFs to the outer mitochondrial membrane (Blümer et al., 2013; Gerondopoulos et al., 2012). Thus, current Rab-targeting models suggest that cytosolic complexes between Rab–GDP and Rab GDP dissociation inhibitor (Rab GDI) continuously and reversibly deliver Rab–GDP to membranes where Rabs are activated by specifically localised GEFs. This blocks their re-extraction by Rab GDI, and promoting accumulation of Rab–GTP in the GEF-associated membrane (Barr, 2013). Related to this, the ‘GEF cascade’ model suggests that the activity of different Rabs in trafficking pathways are linked by sequential recruitment of GEFs. According to this model, active upstream Rabs recruit GEFs of downstream Rabs to membranes as their effectors, thereby regulating the recruitment and activation of downstream Rabs (Ortiz et al., 2002; Rink et al., 2005; Poteryaev et al., 2010; Wang and Ferro-Novick, 2002; Knodler et al., 2010; Novick, 2016).

Rab27 regulates the transport and exocytosis of dense-cored secretory granules and lysosomal-related organelles in many cell types, e.g. pancreatic β cell, cytotoxic T cells and platelets (Fukuda, 2013). In melanocytes, Rab27a targets to the membrane of pigmented melanosomes where active Rab27a–GTP recruits the motor protein myosin-Va via direct interaction with effector melanophilin (Mlph) (Hammer and Sellers, 2012; Hume and Seabra, 2011). This allows actin-dependent dispersion of melanosomes into peripheral dendrites and pigment transfer to neighbouring keratinocytes, thus providing pigmentation and photo-protection in mammals (Wu and Hammer, 2014).

Rab3GEP [also known as MAP kinase-activating death domain protein (MADD), alternatively known as differentially expressed in normal and neoplastic cells (DENN) and insulinoma glucagonoma clone 20 (IG20)] is a GEF for Rab3 whose function is linked to regulated exocytosis and protection against TNFR1 (also known as TNFRSF1A)- and MAPK-driven apoptosis (Wada et al., 1997; Tanaka et al., 2001; Yamaguchi et al., 2002; Imai et al., 2013; Li et al., 2014; Del Villar and Miller, 2004; Kurada et al., 2009). In melanocytes, Rab3GEP promotes melanosome dispersion by acting as a Rab27a GEF and melanosome targeting factor (Figueiredo et al., 2008; Tarafder et al., 2011). Here, we further investigated Rab3GEP function in Rab27a activation and targeting in melanocytes. Our findings indicate that firstly, the DENN domain alone of Rab3GEP is insufficient to activate and target Rab27a to melanosomes; secondly, GEF activity is essential for the Rab27a targeting activity of Rab3GEP; thirdly, Rab3GEP is important, but not alone sufficient, for activation and targeting of Rab27a to melanosomes; and finally, that Rab3GEP is unlikely to stably associate with melanosomes. Based on this we suggest that Rab3GEP differs from other DENN-containing RabGEFs in the mechanism by which it activates Rabs, and that other factors work alongside Rab3GEP to activate and target Rab27a.

## RESULTS AND DISCUSSION

### Melanosome dispersion is partially disrupted in melan-R3G^KO^ melanocytes

**Fig. 1.**
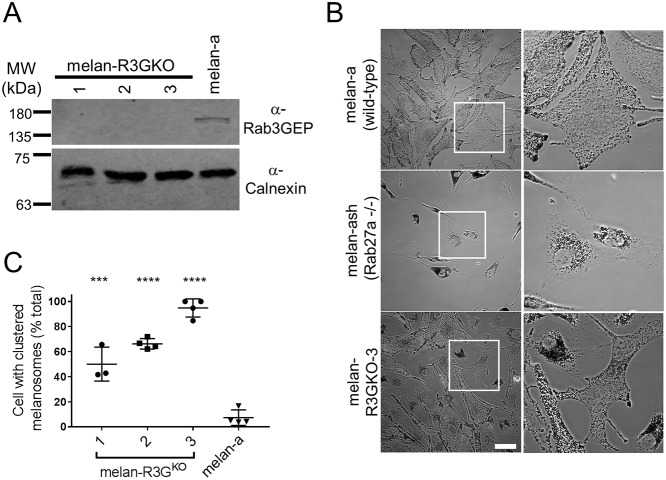
**Melanosomes are dispersed in a sub-set of melan-R3G^KO^ cells.** (A) Western blots comparing Rab3GEP and calnexin expression (loading control) in lysates from melan-R3G^KO1^, melan-R3G^KO2^, melan-R3G^KO3^ and melan-a (wild-type) cell lines. (B) Phase-contrast images showing the distribution of melanosomes in melan-a, melan-ash (Rab27a−/−) and melan-R3G^KO3^ cells. Scale bar: 25 µm. (C) A scatter plot showing the percentage of clustered-type melanocytes in melan-R3G^KO1^, melan-R3G^KO2^, melan-R3G^KO3^ and melan-a cultures 72 h after plating. Data are from 3–4 independent experiments each performed in triplicate. Plotted points represent the mean data in each experiment, data presented as mean±s.e.m. ****P<0.0001, ***P<0.001.

To investigate Rab3GEP function in Rab27a-dependent organelle transport we generated Rab3GEP-deficient immortal melanocyte lines, melan-R3G^KO1^, melan-R3G^KO2^ and melan-R3G^KO3^ (Lavado et al., 2005; Tanaka et al., 2001) (see Materials and Methods). Immunoblotting confirmed that all three melan-R3G^KO^ lines cells lacked detectable levels of Rab3GEP compared with wild-type melanocytes (melan-a) (Fig. 1A). However, in contrast to previous Rab3GEP siRNA knockdown experiments, we found that the three melan-R3G^KO^ cultures contained a mixture of cells in which melanosomes were either dispersed throughout the cytoplasm (as seen in melan-a; hereafter ‘dispersed-type’), or clustered in the perinuclear cytoplasm [as seen in melan-ash (Rab27a−/−) melanocytes; hereafter ‘clustered-type’] (percentage of clustered-type cells 72 h after plating: melan-R3G^KO1^=50.05±7.79, melan-R3G^KO2^=66.22±2.10, melan-R3G^KO3^=94.84±3.62, and melan-a=7.406±6.196; mean±s.e.m.; Fig. 1B,C) (Figueiredo et al., 2008; Tarafder et al., 2011). This indicates that a Rab3GEP-independent mechanism(s) of melanosome dispersion exists in the long-term absence of Rab3GEP. Interestingly, we observed that as melan-R3G^KO^ cells started to proliferate, 48–72 h after plating the proportion of clustered-type cells in cultures increased compared with 24 h after plating (Fig. S1). This suggests that the rate of the Rab3GEP-independent melanosome dispersion pathway(s) does not match the cellular requirement for melanosome dispersion in proliferating cells. Consistent with this, by titration of the essential melanocyte proliferation factor phorbol 12-myristate 13-acetate (PMA) in the culture medium we found that there was a positive correlation between proliferation rate and the proportion of clustered-type melan-R3G^KO^ cells in cultures (Fig. S2). A similar, although smaller, effect was seen in melan-a cells (Fig. S2).

### Melanosome dispersion in melan-R3G^KO^ cells is dependent upon Rab27a

**Fig. 2.**
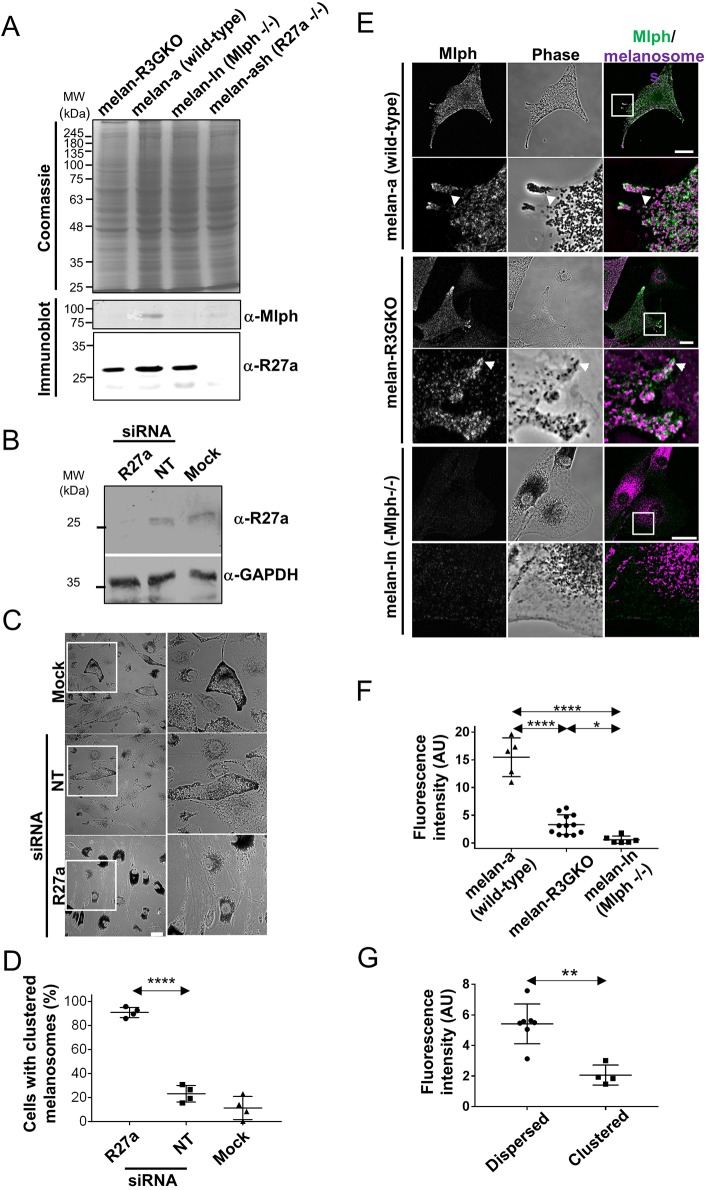
**Rab27a and Mlph disperse melanosomes in melan-R3G^KO^ cells.** (A) Coomassie-stained gel and western blots of cell lysates showing protein loading and Rab27a and Mlph expression. (B–D) melan-R3G^KO^ cells were transfected with Rab27a-specific and non-targeted (NT) control siRNA, and the effect on protein expression and melanosome distribution investigated. (B) Western blot showing Rab27a and GAPDH (loading control) expression in lysates of melan-R3G^KO^ non-transfected (mock) or siRNA-transfected cells. (C) Phase-contrast images showing the melanosome distribution in transfected melan-R3G^KO^ cells. Boxes in left panels indicate the area shown in higher magnification on the right. (D) Scatter plot showing the percentage of clustered-type cells in melan-R3G^KO^ cultures 72 h after transfection. Data are from four independent experiments each performed in triplicate. Plotted points represent the mean data from each experiment, data presented as mean±s.e.m. (E–G) Melanocytes were fixed, stained for immunofluorescence and the distribution of Mlph and melanosomes recorded using a confocal microscope. (E) Fluorescence (left), phase-contrast (centre) and merged images showing the distribution of Mlph, melanosomes and their overlap. White boxes indicate the regions shown in high magnification images below. White arrowheads indicate co-localisation of melanosomes and Mlph. (F,G) Scatter plots showing the mean anti-Mlph fluorescence intensity/cell for different cell types (F), and clustered- and dispersed-type melan-R3G^KO^ cells (G). Results presented are representative of three independent experiments. ****P<0.0001, **P<0.01, *P<0.05. Scale bars: 20 µm.

Next, we tested whether melanosome dispersion in melan-R3G^KO3^ (melan-R3G^KO^ hereafter) cells was Rab27a-dependent. Firstly, we examined the expression of Rab27a and Mlph (whose expression is Rab27a-dependent), in melan-R3G^KO^ and melan-a cells (Hume et al., 2007; Wu et al., 2002). Immunoblotting indicated that expression of both proteins was reduced in melan-R3G^KO^ compared with melan-a cells (Fig. 2A). Secondly, we examined the effect of siRNA depletion of Rab27a on melanosome distribution in melan-R3G^KO^ cells. Immunoblotting and bright-field imaging confirmed that depletion of Rab27a expression in melan-R3G^KO^ cells in resulted in a significant increase in the proportion of clustered-type cells [Fig. 2B–D; non-targeted (NT) siRNA =22.9±3.4%, mock=11.2±4.7%, Rab27a siRNA=90.8±2.1%). Thirdly, expression of GFP–Rab27a efficiently rescued melanosome transport defects in melan-R3G^KO^ cells (Fig. S3). Fourthly, we used confocal microscopy to show that Mlph, a proxy for active Rab27a, was more highly expressed and localised to melanosomes in dispersed-type versus clustered-type melan-R3G^KO^ cells [mean Mlph fluorescence intensity in arbitrary units (AU)/cell: melan-a=11.93±0.96; melan-R3G^KO^=3.31±0.51; melan-ln (Mlph−/−)=0.55±0.41; dispersed-type=5.417±0.5 and clustered-type=2.066±0.33; Fig. 2E–G]. These data support a role for active Rab27a in transporting melanosomes in dispersed-type melan-R3G^KO^ cells, and indicate that Rab27a and Mlph levels are Rab3GEP-dependent.

### The DENN domain is not sufficient for the GEF activity and cellular function of Rab3GEP

**Fig. 3.**
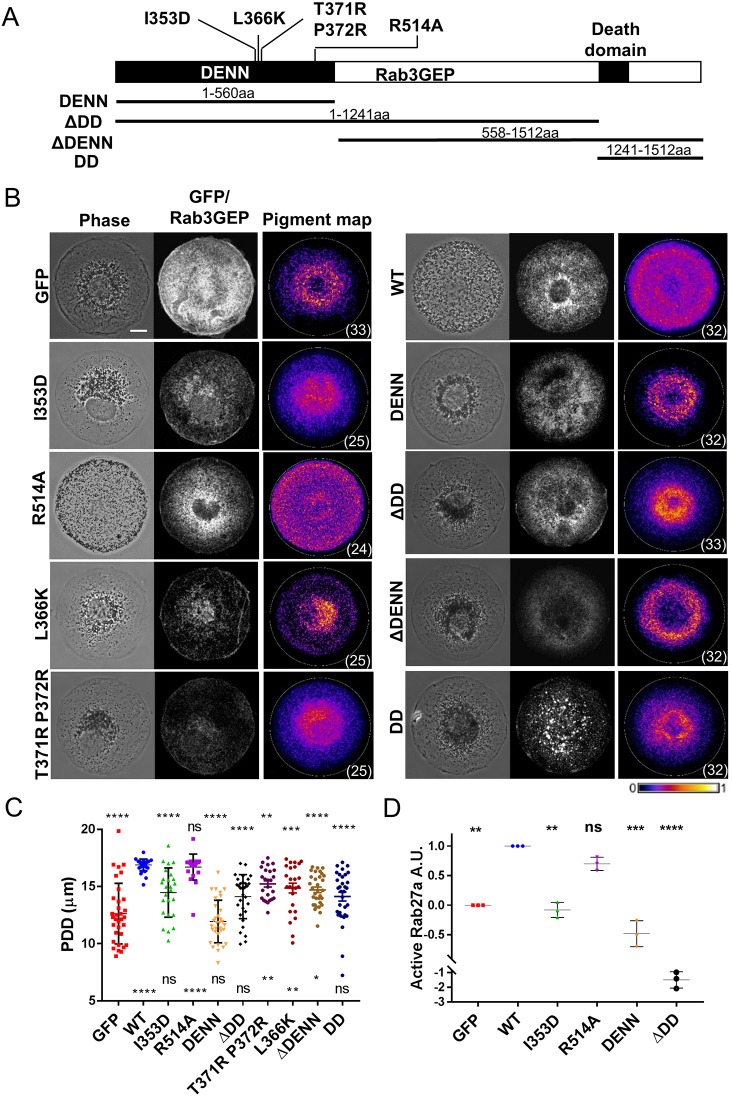
**Rab3GEP-DENN is necessary, but insufficient, for melanosome dispersal in melan-R3G^KO^ cells and Rab27a activation.** (A) A schematic representation of the domain organisation of Rab3GEP (block diagram) showing the position of point mutations. Line diagram indicates the regions included in each of the truncations used in these experiments. (B) melan-R3G^KO^ cells were infected with adenoviruses expressing wild-type (WT) and mutant Rab3GEP, or GFP, plated onto micro-patterned cover-slips, fixed and processed for immunofluorescence. Representative images of melanosome (phase) and GFP (centre) distribution in individual cells expressing the indicated proteins and pigment probability maps (right) for each population of cells (*n* for each indicated in brackets). White circles indicate the shape of the micro-pattern (diameter=46 µm). Scale bar: 10 µm. (C) Scatter plot showing the pigment dispersion distance (PDD) for each cell in each population as shown in B. The significance of differences in PDD values for each mutant compared with the GFP and RabGEP-WT controls was calculated and are displayed below and above each scatter, respectively. (D) Scatter plot showing the ability of Rab3GEP-WT and mutant variants, and GFP alone to activate Rab27a as reported by effector pulldown *in vitro*. Activity is expressed relative to Rab3GEP-WT and GFP controls, as indicated in heatmap key (normalised to 1 and 0). Results are from three independent experiments. The significance of differences in active Rab27a values for each mutant and GFP compared with RabGEP-WT are above each scatter. Data presented as mean±s.e.m. ****P<0.0001; ***P<0.001; **P<0.01; ns, not significant.

We then used the melan-R3G^KO^ cells to dissect the role of Rab3GEP domains in Rab27a targeting and activation. To test whether the DENN domain of R3G is sufficient for GEF activity, as seen in other DENN-containing RabGEFs, we generated a model of Rab3GEP-DENN based on the structure of the DENND1B–Rab35 complex (Allaire et al., 2010; Wu et al., 2011; Ioannou et al., 2015) (Fig. S4A; see Materials and Methods). Using this model, we identified residues in Rab3GEP-DENN that could interact with Rab27a, and generated vectors expressing mutants expected to disrupt this interaction in melanocytes (Rab binding site I: I353D or L366K, and site II: T371R/P372R) (Fig. 3A; Fig. S4B). [To help quantify Rab3GEP function in melanosome dispersion we standardised melanocyte shape by growing the cells on fibronectin micro-patterns (Evans et al., 2014).] In melan-R3G^KO^ we found that Rab3GEP^I353D^ and other mutants dispersed melanosomes to an intermediate level compared with GFP-only control and wild-type Rab3GEP (Rab3GEP-WT) [pigment dispersion distance (PDD): Rab3GEP^I353D^=14.47±2.156 µm; Rab3GEP^L366K^=14.87±2.121 µm; Rab3GEP^T371R/P372R^=15.23±1.36 µm; Rab3GEP-WT=16.89±0.5225 µm and GFP=12.63±2.648 µm; Fig. 3B,C]. In contrast, the Rab3GEP^R514A^ mutant, in which the altered residue is outside the predicted Rab binding sites (Fig. S4B), rescued melanosome dispersion in melan-R3G^KO^ cells with similar efficiency to Rab3GEP-WT (PDD: 16.71±1.138 µm; Fig. 3A–C). These data support the importance of the Rab3GEP DENN domain, and suggest that the interaction mechanism of DENN-containing GEFs with Rabs is conserved.

To better understand how DENN mutants reduced cellular Rab3GEP function, we tested their effect on Rab3GEP GEF activity using an effector pulldown assay that reports Rab27a–GTP levels (see Materials and Methods) (Figueiredo et al., 2008). In accord with the results of the melanosome dispersion assay we saw that the Rab27a–GTP levels in Rab3GEP^I353D^ and Rab3GEP^R514A^ were similar to those seen in cells expressing GFP and Rab3GEP-WT, indicating that GEF activity is essential for Rab3GEP function in melanosome transport and that the DENN domain plays an important role in both of these activities (Fig. 3D).

We next used a DENN alone truncation to test whether Rab3GEP-DENN catalysed Rab27a-specific GEF activity and Rab27a-dependent melanosome dispersion. Using both the cell and pulldown assays we found that Rab3GEP^DENN^ functioned with comparable efficiency to GFP and significantly less efficiently than Rab3GEP-WT (PDD: 11.94±1.859 µm; Fig. 3B–D). Similar results were obtained using the Rab3GEP^ΔDD^ mutant that lacks the C-terminus death domain (DD) (PDD=14.12±1.922 µm; Fig. 3A–C). These observations, with others using N-terminus Rab3GEP truncation mutants, indicate that while the DENN is important, other parts of the protein, including the DD and central region, are also required for Rab3GEP function (PDD: ΔDENN=14.71±1.369 µm; ΔDD=14.13±2.257 µm; Fig. 3A–C). As previously seen, Rab3GEP and variants were distributed throughout the melanocyte cytoplasm (even at very low expression levels) and not enriched near melanosomes (Fig. 3B; Fig. S4C) (Figueiredo et al., 2008). This indicates that Rab3GEP is unlikely to stably associate with melanosomes. The exception to this was the Rab3GEP^DD^ mutant, which distributed in cytoplasmic punctae, consistent with the ability of DDs to oligomerise (Feinstein et al., 1995) (Fig. 3B; Fig. S4C). Results were similar for Rab3GEP and mutants in non-pattern grown cells (Fig. S4C).

### Intact mitochondria-localised Rab3GEP targets Rab27a to mitochondria

**Fig. 4.**
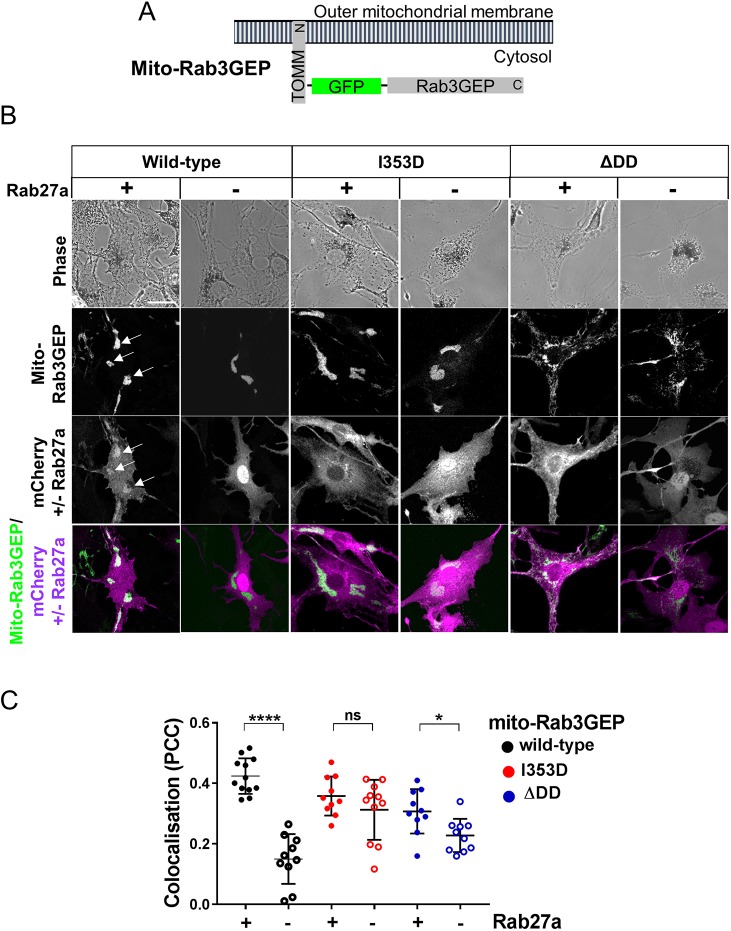
**Mito-Rab3GEP can re-target Rab27a to mitochondria by a GEF-dependent mechanism.** (A) Schematic representation of mito-Rab3GEP showing the arrangement of proteins within the fusion and the topology of its association with the outer mitochondrial membrane. (B) melan-R3G^KO^ cells were co-infected with adenoviruses expressing GFP-tagged mito-Rab3GEP (wild-type) or mutants (I353D and ΔDD), and mCherry–Rab27a or mCherry, then fixed and protein distribution recorded by confocal microscopy. Co-localisation of GFP and mCherry was determined using Pearson correlation analysis. Confocal images show the distribution of melanosomes (phase), mito-Rab3GEP and mutants, mCherry–Rab27a or mCherry, and their co-localisation. Scale bar: 20 µm. (C) Scatter plot showing the extent of colocalisation of (1) mito-Rab3GEP and mutant variants and (2) mCherry–Rab27a (+, solid circles) or mCherry (−, open circles). The significance of differences between Pearson correlation co-efficient values for populations expressing mCherry versus mCherry–Rab27a for each mito-Rab3GEP protein were calculated using an unpaired Student's *t*-test. ****P<0.0001; *P<0.05; ns, not significant.

Finally, we investigated the role of Rab3GEP in targeting Rab27a to organelle membranes. For this we generated a fusion protein that targeted Rab3GEP-WT to the outer mitochondrial membrane (mito-Rab3GEP) and tested its ability to target mCherry–Rab27a to mitochondria in melan-R3G^KO^ cells (Fig. 4A). Mito-Rab3GEP localised to MitoTracker-labelled mitochondria but did not disperse melanosomes (Fig. 4B; Fig. S3). In most cells we saw that mito-Rab3GEP-positive mitochondria aggregated close to the nucleus, in contrast to their normal dispersed cytoplasmic distribution, suggesting that mito-Rab3GEP causes their aggregation. We also saw that mCherry–Rab27a co-localised with mito-Rab3GEP to a significantly greater extent than mCherry alone. (PCC: mito-Rab3GEP/mCherry–Rab27a=0.424±0.016 versus mito-Rab3GEP/mCherry=0.149±0.026; Fig. 4B,C). This indicates that Rab3GEP influences Rab27a localisation and that tethering Rab3GEP to mitochondria reduces its ability to activate and target Rab27a to melanosomes.

We then examined the importance of the GEF activity of Rab3GEP in Rab27a targeting. We found that GEF-deficient mito-Rab3GEP^I353D^ and mito-Rab3GEP^ΔDD^ mutants localised to mitochondria, but recruited mCherry–Rab27a to a significantly lower extent than wild-type mito-Rab3GEP (PCC: mito-Rab3GEP^I353D^/mCherry–Rab27a=0.358±0.020; mito-Rab3GEP^I353D^/mCherry=0.312±0.029; mito-Rab3GEP^ΔDD^/mCherry–Rab27a=0.307±0.023; mito-Rab3GEP^ΔDD^/mCherry=0.227±0.017; Fig. 4B,C). This indicates that the GEF activity of Rab3GEP is required for Rab27a targeting. We observed that mitochondria in cells expressing mito-Rab3GEP^ΔDD^, but not other mito-Rab3GEPs, were dispersed throughout the cytoplasm, indicating that DD promotes mitochondrial aggregation, possibly through oligomerisation (Feinstein et al., 1995).

Here, we investigated how Rab3GEP regulates Rab27a activation and/or targeting using melanocytes as a model and present several novel findings. Firstly, Rab27a can undergo Rab3GEP-independent activation and/or targeting, and that although Rab3GEP enhances these activities it is not absolutely required. One possible mechanism for this is that other GEFs compensate for the loss of Rab3GEP, e.g. DENND4B and GRAB (also known as Rab3IL1) (Figueiredo et al., 2008; Yoshimura et al., 2010). However, siRNA depletion of these targets did not augment the proportion of clustered-type cells seen in melan-R3G^KO^ cells, indicating that these GEFs do not contribute to Rab27a activation (Fig. S4A,B). Another possible mechanism is that the intrinsic nucleotide exchange activity of Rab27a disperses melanosomes in slow-growing cells. Supporting this, *in vitro* studies of GTP loading of purified Rabs indicate that Rab27a has a higher intrinsic nucleotide exchange activity compared with Rab1 and Rab5a that reaches ∼35% of the level achieved in the presence of purified Rab3GEP (Fig. S4C). This, coupled with the low rate of intrinsic Rab27a GTPase activity (one thirtieth that of Rab5a), could explain the existence of a pool of active Rab27a in melan-R3G^KO^ cells (Larijani et al., 2003). Interestingly a yeast Rab7 mutant (Ypt7^K127E^), that has been shown to enhance nucleotide exchange and reduce nucleotide affinity, targeted to vacuolar membranes in the absence of its GEF, the Mon1–Ccz1 complex (Cabrera and Ungermann, 2013). These data underline that RabGEFs are enhancers rather than absolute determinants of Rab activation and/or targeting. Thus, it is likely that the severe phenotypic alterations seen in Rab3GEP-deficient mice and worms result from reduced function of their multiple Rab substrates (Iwasaki et al., 1997; Tanaka et al., 2001). Secondly, Rab3GEP-DENN is necessary, but insufficient, to catalyse Rab27a activation and/or targeting. This contradicts studies showing that DENN-only truncations of connecdenn (also known as DENND1B) and DENND2B maintained GEF activities comparable with their intact counterparts (Allaire et al., 2010; Wu et al., 2011; Ioannou et al., 2015). In contrast, our data indicate that elements throughout Rab3GEP make significant contributions to its activity. Studies using Rab3a as a Rab3GEP substrate reached similar conclusions (Oishi et al., 1998; Coppola et al., 2002). Meanwhile, sequence comparison indicates that there is significant conservation of DENN and non-DENN elements of Rab3GEP throughout evolution (Iwasaki et al., 1997; Mahoney et al., 2006). Thus, Rab3GEP likely activates and/or targets Rab27a and Rab3a via a similar mechanism, but this may differ from that of other DENN-containing RabGEFs e.g. connecdenn, DENND1A and DENND2B. Thirdly, the GEF activity of Rab3GEP is absolutely required for its function in targeting Rab27a to membranes. These observations are consistent with previous work showing that the membrane-targeting activity of Rabex-5 (also known as RABGEF1), DrrA and Rabin8 (also known as RAB3IP) was dependent upon their GEF activity (Blümer et al., 2013). Fourthly, we show that the cytoplasmic localisation of Rab3GEP is important for its function in Rab27a targeting and/or activation.

In conclusion, our data are broadly consistent with models suggesting that RabGEFs serve as important targeting factors by locally activating Rabs and stabilising their association with membranes by preventing their extraction by Rab GDI. Nevertheless, our findings here and previously indicate that Rab3GEP is unlikely to be the sole Rab27a targeting factor (Tarafder et al., 2011). Future work should aim to identify and characterise these factors.

## MATERIALS AND METHODS

### Derivation and maintenance of immortal melanocytes

Cultures of immortal Rab3GEP-deficient melanocytes (melan-RG3^KO^) were derived essentially as described previously (Lavado et al., 2005). In brief, mice heterozygous for a previously generated Rab3GEP loss-of-function allele were crossed with Ink4a-Arf mutant mice in order to generate embryos homozygous for the Rab3GEP mutant allele and heterozygous for Ink4a-Arf mutant allele. Genotyping of the embryos was as previously described (Lavado et al., 2005; Tanaka et al., 2001). Melanocytes were then derived from the dorsal skin of mutant embryos as previously described (Bennett et al., 1989). melan-R3G^KO1^, melan-R3G^KO2^ and melan-R3G^KO3^ melanocytes were derived from three different embryos from the same litter. Cultures of immortal melan-RG3^KO^, melan-ash, melan-a and melan-ln melanocytes were maintained, infected with adenovirus expression vectors, transfected with siRNA oligonucleotides and tested for contamination as described previously (Hume et al., 2006, 2007). The melanocyte cell lines described here are available from the Wellcome Trust Functional Genomics Cell Bank (http://www.sgul.ac.uk/depts/anatomy/pages/WTFGCB.htm).

### Immunoblotting

Immunoblotting was performed as described previously (Hume et al., 2007) using rabbit anti-Rab3GEP (gift from Dr Yoshimi Takai, Osaka University Graduate School of Medicine, Japan; 1:1000) goat anti-melanophilin (Everest Biotech, EB05444; 1:1000), goat anti-GAPDH (Sicgen, Ab0049-200; 1:5000), goat anti-calnexin (Sicgen, Ab3741-200; 1:1000) and goat anti-Rab27 (Sicgen, Ab1023-200; 1:1000) primary antibodies, and IRDye 800CW-conjugated secondary antibodies (Odyssey 926-32214; 1:10,000). Signal was detected using a Li-Cor infrared scanner (Odyssey).

### Plasmid and virus constructs

Generation of virus vectors allowing expression of full-length human Rab3GEP as a fusion to the C-terminus of EGFP was previously described (Figueiredo et al., 2008). Adenoviruses expressing Rab3GEP^I353D^, Rab3GEP^R514A^, Rab3GEP^L366K^, Rab3GEP^T371R/P372R^, Rab3GEP^ΔDD^, Rab3GEP^ΔDENN^, Rab3GEP^DENN^ and Rab3GEP^DD^ were all generated using quick-change site-directed mutagenesis, using pENTR-GFP-Rab3GEP as template (the sequences of primers used for this are available on request). To generate adenoviruses expressing mito-Rab3GEP we PCR-amplified a 240 bp fragment of DNA corresponding to the N-terminus mitochondrial targeting sequence of murine TOMM70a (GenBank accession number AAI39422.1). This was then ligated into an engineered HindIII site located upstream of the 5′ end of the EGFP coding sequence of pENTR-GFP-Rab3GEP. Mutants variants of this were generated by site-directed mutagenesis as described above.

### Microscopy and image analysis

Cells for immunofluorescence were cultured on 13 mm coverslips (1.5 thickness; Scientific Laboratory Supplies UK, 6422-307164), paraformaldehyde fixed, stained, and fluorescence and transmitted light images of melanocytes were then collected using a Zeiss LSM710 confocal microscope fitted with a 63×1.4 NA oil immersion Apochromat lens or a Zeiss Axiovert 100S inverted microscope fitted with a 10× objective and an Axiocam MR3 CCD camera. All images presented are single sections in the *z*-plane. Antibodies and stains were used as indicated; mouse monoclonal anti-GFP (Roche, 11814460001; 1:200) rabbit anti-Mlph [antigen mouse Mlph 150–400 aa (Strom et al., 2002); 1:100] goat anti-rabbit and goat anti-mouse IgG secondary antibodies both Alexa Fluor 568 labelled (Invitrogen, A-11001 and A-11011; both 1:500). For live-cell experiments confirming the targeting of mito-Rab3GEP to mitochondria, cells were plated in 35 mm diameter glass-bottomed petri dishes (Matek, P35G-1.5-20-C) (1×104 cells/dish). 24 h later cells were infected with adenoviruses expressing mito-Rab3GEP or GFP alone, and after a further 48 h the mitochondria were labelled by incubation for 30 min in 200 nM MitoTracker Red FM (Thermo Fisher, M7512). After washing twice in medium (L-15 supplemented with 10% fetal calf serum, 100 U/ml penicillin G, and 100 mg/ml streptomycin) without MitoTracker, cells were transferred to the environmental chamber (37°C) surrounding the stage of the Zeiss LSM710 confocal microscope and images of the distribution of GFP, MitoTracker-labelled mitochondria and melanosomes were acquired using the 488 nm Ar and 568 nm HeNe laser lines, respectively. Analysis of melanosome clustering in siRNA-transfected cells and melanosome distribution in micro-pattern grown cells was as previously described (Evans et al., 2014; Robinson et al., 2017).

### Effector pulldown assay for detection of Rab27a–GTP

HEK293a cells were plated in 10 cm dishes (∼5×10^6^ cells/dish) and 24 h later co-infected with adenoviruses expressing mCherry–Rab27a and either GFP, Rab3GEP-WT or mutant variants of Rab3GEP. After expression for 24 h cells were washed twice with ice-cold PBS and lysed using buffer A [50 mM Tris pH 7.5, 150 mM NaCl, 5 mM MgCl_2_, 1 mM dithiothreitol, 1% CHAPS, and protease inhibitor cocktail (Complete ULTRA Tablets, Roche)] for 30 min on ice. Cell contents were harvested using a scraper and lysates were clarified by centrifugation at 13,400 ***g*** for 15 min at 4°C using Eppendorf centrifuge 5415R. Supernatant was collected and protein quantification was performed using a Bradford protein assay (Bio-Rad). 2 mg of the total protein was pre-cleared by incubation with 20 µl of glutathione-sepharose 4B Fast Flow beads (GE Healthcare, 17-5132-01) for 2 h. After pelleting the beads by centrifugation at 3220 ***g*** at 4°C the supernatant was incubated with 200 pmol of GST or 200 pmol of GST–Slp1 (1–200 aa fragment) and 25 µl of glutathione-sepharose 4B beads for 16 h at 4°C. Beads were pelleted as above and washed three times in buffer A 10 min. Bound proteins were released from the beads by incubation in Laemmli buffer [100 mM Tris-Cl pH 6.8, 4% (w/v) SDS, 0.2% (w/v) Bromophenol Blue, 20% (v/v) glycerol and 200 mM β-mercaptoethanol] for 5 min at 95°C. Bound proteins were then resolved by SDS-PAGE and mCherry–Rab27a was detected by immunoblotting and signal intensity quantified using ImageJ.

### Modelling of the structure of the DENN domain of Rab3GEP

The structural model of Rab3GEP was produced using the HHpred server in conjunction with MODELLER (Alva et al., 2016). In brief, the amino acid sequence of the GEF domain of Rab3GEP was submitted to HHpred first. The server identified the GEF domain of DENND1A as the closest structural homologue. Subsequently, the generated amino acid sequence alignment between Rab3GEP and DENND1A was used as a basis for producing a structural model of Rab3GEP using MODELLER by directly forwarding the result produced through HHpred.

### Quantitative real-time PCR

Primers and probes for qRT-PCR targets (from Sigma Genosys, Cambridge, UK) were designed using Primer Express software (Life Technologies). Probes were labelled at the 5′ and 3′ ends with fluorophore 6-carboxyfluorescein (6-FAM) and quencher tetramethylrhodamine (TAMRA), respectively. For GRAB and DENND4B the primers were 5′-CAGCCTGTTTGAGGAAGCTC-3′ (sense strand) and 5′-TGGTGTGGATGTGATGACCA-3′ (reverse strand), and 5′-TGCGCCACGTCGGACTCAAC-3′ (sense strand) and 5′-TCCTTGCCCATGCTGCTGGC-3′ (reverse strand) and the probes were 5′- CGCCTGCTTCATGTTGGCTTCCCG-3′ and 5′-GAGACGCTAGGGCCCCCTCC-3′. For GAPDH the primers were 5′-GTGTCCGTCGTGGATCTGA-3′ (sense strand) and 5′-CCTGCTTCACCACCTTCTTGA-3′ (reverse strand) and the probe was 5′-CCGCCTGGAGAAACCTGCCAAGTATG-3′. To generate mRNA, sample pools of melan-R3G^KO^ cells grown in 6-well plates (1×10^5^ cells/well) were transfected with siRNA in triplicate as described previously (Hume et al., 2007). 72 h later cells were harvested and mRNA extracted using the RNeasy Mini RNA extraction kit (Qiagen). cDNA was generated using Moloney murine leukemia virus M-MLV reverse transcriptase (Promega) using random primers. To generate a standard curve of signal:template concentration for each qRT-PCR assay, a pool containing 5% of each of the cDNA samples analysed was generated. This pool was serially diluted in DEPC water (1:4, 1:16, 1:64, 1:256) and these were used as template in Rab1a and GAPDH qRT-PCR assays. The shape of the standard curve indicates the relationship between signal and template concentration. For both assays standard curves gave straight lines with R^2^>0.99 indicating that there is a linear relationship between signal and template. To measure the expression of targets in siRNA-transfected cells, each neat cDNA was diluted 1:32 in DEPC water and the following reagents were added per well of a 96-well plate: 6.5 µl TaqMan Fast 2× PCR Master Mix (Life Technologies); 0.4 µl forward primer (10 µM); 0.4 µl reverse primer (10 µM); 0.25 µl probe (10 µM); 3 µl cDNA; 2.45 µl DEPC water. For each sample, three technical repeats were performed. Reaction plates were sealed with optically clear adhesive film, centrifuged, and qRT-PCR performed using a StepOnePlus Real-Time PCR system (Applied Biosystems) using the ‘fast’ mode. CT values for each reaction were determined by StepOne software. The slope (S), intercept (I) and R^2^ values were calculated for the standard curve of each qRT-PCR assay. CT values from siRNA-transfected samples were then processed to generate a ‘quantity value’ for each CT value as follows; (1) (CT−I)/S=LQ (LQ, log quantity), (2) 10LQ=Q, (3) Q×(1/MNT)=GOIP (MNT, mean non-targeted quantity value; GOIP, gene of interest product) and (4) GOIP/GP=normalised expression of target relative to GAPDH (GP, normalised quantity value for the GAPDH primer).

### Statistical analysis of data

Unless otherwise indicated, statistical analysis of data was carried out with GraphPad Prism 7 software using the one-way ANOVA test and Bonferroni's multiple comparisons post-test facility within the software and assuming nonparametric distribution of data. ****P<0.0001, ***P<0.001, **P<0.01, *P<0.05 and ns, not significant (*P*>0.05).

## Supplementary Material

Supplementary information
