# Supplementary material for: Nucleotide exchange factor Rab3GEP requires DENN and non-DENN elements for activation and targeting of Rab27a
Source: J Cell Sci. 2019 Apr 30;132(9):jcs212035. doi: 10.1242/jcs.212035 (PMC6526710; doi:10.1242/jcs.212035)
Supplement: Supplementary information [file joces-132-212035-s1.pdf]

Figure S1. Proliferation rate correlates positively with defects in melanosome dispersion in melan-R3G^{KO} cells. (A-C) melan-R3G^{KO} and melan-a cells were seeded into 24-well tissue culture plates (1.5x10^{4} cells/well) and the numbers of (A, D), and proportion of clustered- versus dispersed-type cells (B-C, E-F) was recorded over time (as described in materials and methods). (A-C) cells cultured in 40nM PMA and (D-F) either 0 or 80nM PMA. (A-B, D-E) Line plots and (C, F) phase contrast images showing the rate of proliferation, and proportion of clustered-type melanocytes in cultures of melan-a and melan-R3G^{KO}. Red and black arrows indicate dispersed- and clustered-type melan-R3G^{KO} melanocytes, respectively. Scale bar = 100μm. Results are representative of 3 independent experiments.

Journal of Cell Science • Supplementary information

Figure S1

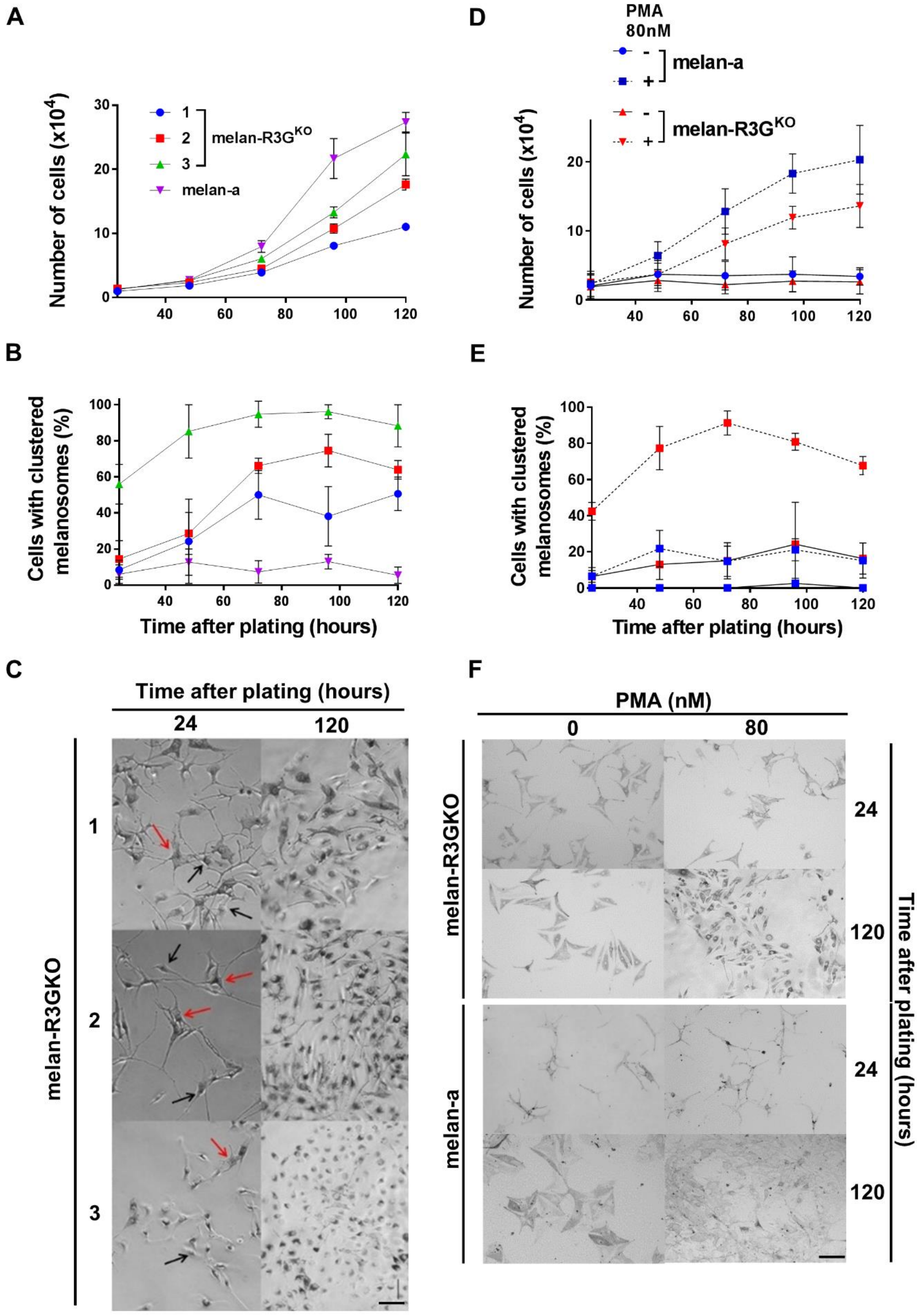

Journal of Cell Science • Supplementary information

Figure S2. Expression of GFP-Rab3GEP or GFP-Rab27a, but not Rab3GEP-DENN domain mutants, rescues defects in melanosome dispersion in melan-R3G^{KO} cells. A) Cover-slip grown melan-R3G^{KO} cells were infected with adenoviruses expressing the indicated GFP fusion protein, fixed 24 hours later and the distribution of melanosomes and GFP protein was recorded using a microscope. Images showing the distribution of melanosomes (bright-field) and GFP fusion distribution Scale bar = 50 μm. (B) A structural model of the DENN domain of Rab3GEP was generated based on the previously determined structure of DENND1A (as described in materials and methods). Ribbon diagrams of the 3D structure of the DENN domains of DENND1A and Rab3GEP individually and overlaid to allow comparison (Wu et al 2011). (C) Space filling and ribbon diagrams (upper and lower panels, respectively) indicating the position of Rab binding sites I and II (left- and right-hand sides) in the DENN domains (red in DENND1A and blue in Rab3GEP). Upper and lower panels show the location of site I and II in the context of the entire DENN (as well as the location of R514), and the position of residues mutated in each of these in this study, respectively. White boxes in lower panels indicate the correspondence between site I and II mutations in DENND1B and Rab3GEP. (D) To test the functional importance of the predicted Rab27a binding sites in DENN and other Rab3GEP domains, melan-R3G^{KO} cells were infected with adenoviruses expressing GFP-tagged of the indicated Rab3GEP proteins. Cells were then fixed, processes for immunofluorescence and the distribution of melanosomes (using transmitted light/phase contrast optics) and GFP was examined using a confocal microscope (as described in material and methods). White arrows indicate cells with dispersed melanosome distribution. Scale bar = 50 μm (A) and 20 μm (D).

Journal of Cell Science • Supplementary information

Figure S2

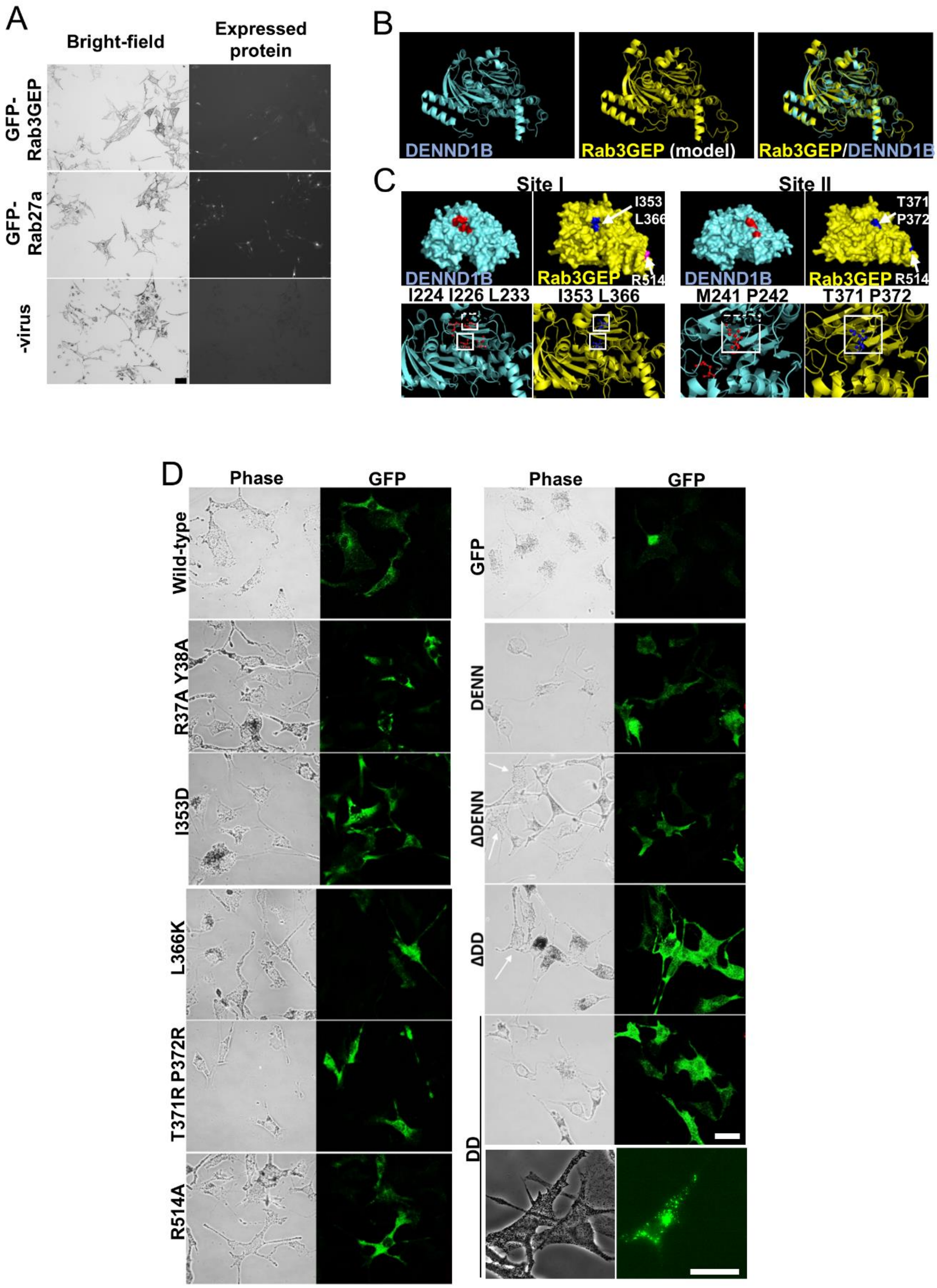

Journal of Cell Science • Supplementary information

Figure S3. Mito-Rab3GEP targets to mitochondria and does not disperse melanosomes in melan-R3G^{KO} cells. (A) melan-R3G^{KO} cells grown in glass-bottomed culture dishes were infected with adenoviruses expressing mito-Rab3GEP or GFP, labelled 48 hours later with mito-tracker red to reveal the distribution of mitochondria and images alive using a confocal microscope (as described in materials and methods). Images show the distribution of mito-Rab3GEP/GFP, mitotracker and the extent of their overlap (merge) and the distribution of melanosomes (phase contrast) Scale bar = 10μm. (B-C) melan-R3G^{KO} cells were infected with adenoviruses expressing mito-Rab3GEP or Rab3GEP-WT, fixed 48 hours later, imaged using bright-field and epifluorescence optics to reveal the distribution of melanosomes in populations of cells and confirm the expression of the Rab3GEP proteins (B). The proportion of clustered-type melanocytes was determined for each condition (C). Data are from 4 independent experiments each performed in triplicate on different pools of cells. Plotted points represent the average percentage of cells with perinuclear clustered melanosomes from each experiment (as described in material and methods). **** indicate p=<0.0001, relative to Rab27a depleted cells was determined by unpaired student's t-test.

Journal of Cell Science • Supplementary information

Figure S3

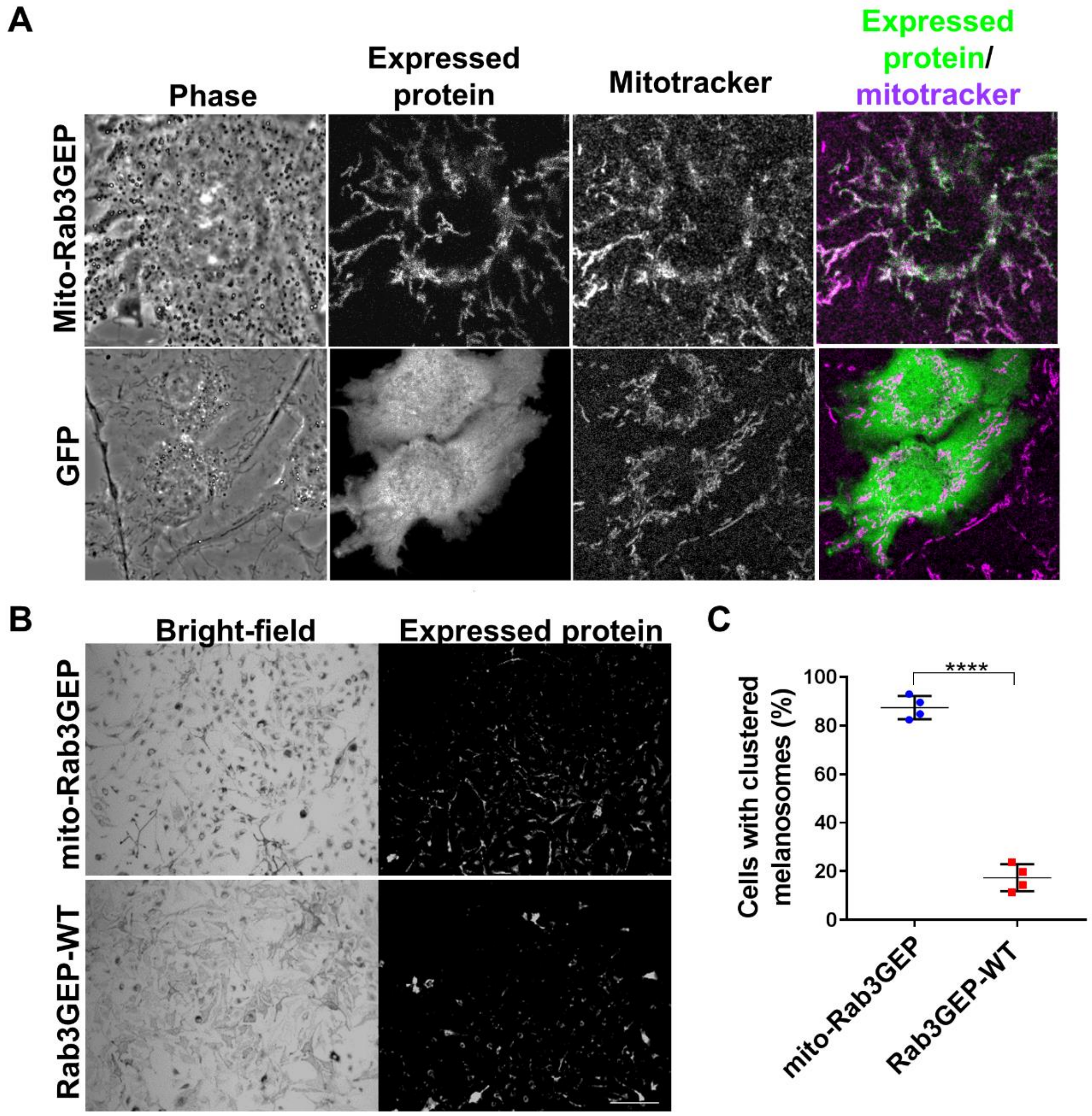
A
Phase
Expressed protein
Mitotracker
Expressed protein/ mitotracker
Mito-Rab3GEP
GFP
B
Bright-field
Expressed protein
mito-Rab3GEP
Rab3GEP-WT
C
Cells with clustered melanosomes (%)
100
80
60
40
20
0
****
mito-Rab3GEP
Rab3GEP-WT

Figure S4. The intrinsic GDP/GTP exchange activity in vitro Rab27a, rather than alternative GEFs, is likely to be responsible for the phenotype of dispersed-type melan-R3G^{KO} cells. (A) Purified $(His)_6$-Rab1a, $(His)_6$-Rab5a and $(His)_6$-Rab27a were incubated with [35S]-GTPγS at 30°C for the indicated time periods in the absence of Rab3GEP. [35S]-GTPγS binding was quantified by filter binding assay followed by scintillation counting as previously described (Figueiredo et al., 2008). (B-C) melan-R3G^{KO} cells were transfected with the indicated siRNA (as described in Materials and methods) and the effects of this on mRNA levels (B) melanosome distribution (C) and was investigated. Data are from at least 3 independent experiments each performed in triplicate on different pools of cells. Plotted points represent the average percentage of cells with perinuclear clustered melanosomes from each experiment (as described in material and methods). **** and ** indicate p=<0.0001 and p=<0.01, relative to NT siRNA transfected cells was determined by one-way ANOVA. Scale bar = 50μm.

Journal of Cell Science • Supplementary information

Figure S4

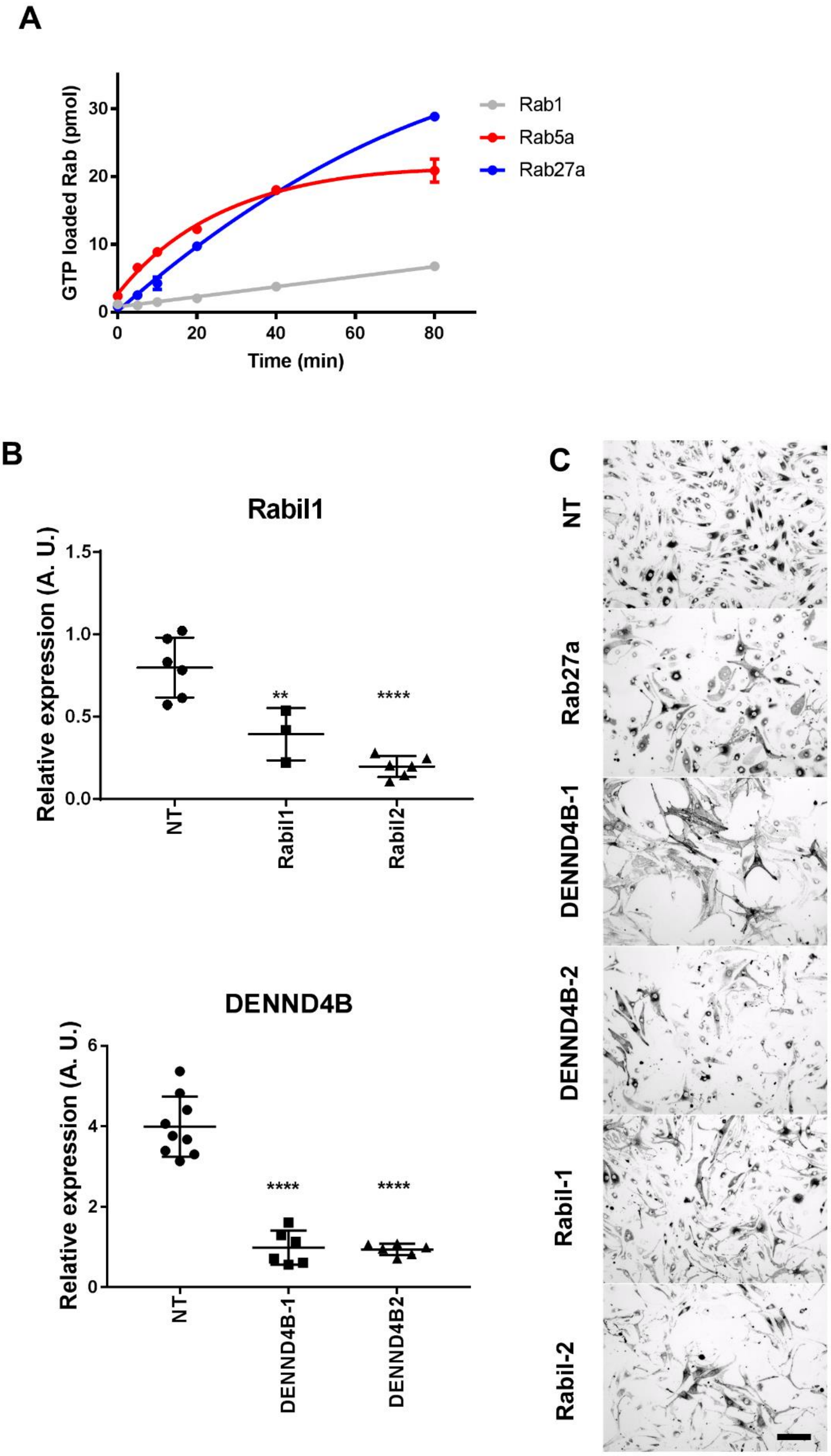

Journal of Cell Science • Supplementary information

References.

FIGUEIREDO, A. C., WASMEIER, C., TARAFDER, A. K., RAMALHO, J. S., BARON, R. A. & SEABRA, M. C. 2008. Rab3GEP is the non-redundant guanine nucleotide exchange factor for Rab27a in melanocytes. *J Biol Chem,* 283**,** 23209-16.
